# Functional and proteomic analysis of a full thickness filaggrin-deficient skin organoid model

**DOI:** 10.12688/wellcomeopenres.15405.2

**Published:** 2019-11-26

**Authors:** Martina S. Elias, Sheila C. Wright, William V. Nicholson, Kimberley D. Morrison, Alan R. Prescott, Sara Ten Have, Phillip D. Whitfield, Angus I. Lamond, Sara J. Brown

**Affiliations:** 1 Skin Research Group, Division of Molecular and Clinical Medicine, School of Medicine, University of Dundee, Dundee, Scotland, DD1 9SY, UK; 2 Dundee Imaging Facility, School of Life Sciences, University of Dundee, Dundee, Scotland, DD1 5EH, UK; 3 Centre for Gene Regulation and Expression, School of Life Sciences, University of Dundee, Dundee, Scotland, DD1 5EH, UK; 4 Lipidomics Research Facility, Division of Biomedical Sciences, University of the Highlands and Islands, Inverness, Scotland, IV2 3JH, UK; 5 Department of Dermatology, Ninewells Hospital, Dundee, Scotland, DD1 9SY, UK

**Keywords:** Axon guidance, atopic dermatitis, eczema, filaggrin, gene ontology, keratinocyte-immune crosstalk, organoid, proteomics

## Abstract

**Background:** Atopic eczema is an itchy inflammatory disorder characterised by skin barrier dysfunction. Loss-of-function mutations in the gene encoding filaggrin ( *FLG*) are a major risk factor, but the mechanisms by which filaggrin haploinsufficiency leads to atopic inflammation remain incompletely understood. Skin as an organ that can be modelled using primary cells *in vitro *provides the opportunity for selected genetic effects to be investigated in detail.

**Methods:** Primary human keratinocytes and donor-matched primary fibroblasts from healthy individuals were used to create skin organoid models with and without siRNA-mediated knockdown of *FLG*. Biological replicate sets of organoids were assessed using histological, functional and biochemical measurements.

**Results:** *FLG *knockdown leads to subtle changes in histology and ultrastructure including a reduction in thickness of the stratum corneum and smaller, less numerous keratohyalin granules. Immature organoids showed some limited evidence of barrier impairment with *FLG *knockdown, but the mature organoids showed no difference in transepidermal water loss, water content or dye penetration. There was no difference in epidermal ceramide content. Mass spectrometry proteomic analysis detected >8000 proteins per sample. Gene ontology and pathway analyses identified an increase in transcriptional and translational activity but a reduction in proteins contributing to terminal differentiation, including caspase 14, dermokine, AKT1 and TGF-beta-1. Aspects of innate and adaptive immunity were represented in both the up-regulated and down-regulated protein groups, as was the term ‘axon guidance’.

**Conclusions: **This work provides further evidence for keratinocyte-specific mechanisms contributing to immune and neurological, as well as structural, aspects of skin barrier dysfunction. Individuals with filaggrin deficiency may derive benefit from future therapies targeting keratinocyte-immune crosstalk and neurogenic pruritus.

## Introduction

Atopic eczema (also termed atopic dermatitis or eczema ^1, 2^) is an itchy inflammatory skin disorder with complex and multifactorial aetiology ^3^. The phenotype is heterogeneous and pathogenic mechanisms vary between affected individuals ^4^. The pathogenesis may include differential contributions of: immune activation and skin barrier dysfunction; local and systemic effects; multiple genetic and environmental factors ^5^. Loss-of-function mutations in *FLG*, encoding filaggrin, cause the common Mendelian disorder of keratinization, ichthyosis vulgaris (OMIM # 146700). *FLG* null mutations are also strongly associated with increased risk of atopic eczema ^6, 7^ and multiple other atopic traits ^8– 10^.

Skin is an organ that can be modelled *in vitro* to effectively recapitulate the multi-layered structure and gene expression patterns of human skin *in vivo* ^11^. This offers the opportunity for selected molecular mechanisms to be investigated in relative isolation from a complex disease state, using relevant primary cells. Replicate experiments can be performed using primary cells with the same genetic background or cells from different donors ^12^. Detailed functional and biochemical analyses may then be applied to define potential therapeutic targets ^12, 13^.

Filaggrin is a marker of keratinocyte differentiation; it is expressed in the granular layer of the upper epidermis as a polymer – profilaggrin – which undergoes post-translational modification and stepwise proteolysis to release monomeric filaggrin ^14^. Profilaggrin and filaggrin have multiple functions, each of which have been described as contributing to the mechanical, biochemical, immunological and microbiological aspects of skin barrier function ^15^. Clinical studies have shown that filaggrin haploinsufficiency is associated with xerosis and ichthyosis (dry and scaly skin), keratosis pilaris (increased keratin within skin follicles), palmar hyperlinearity ^16^ and increased transepidermal water loss ^17^. Transcriptomic analysis of full thickness skin biopsies have shown evidence of an abnormal defence response in *FLG* haploinsufficient atopic skin ^18^; proteomic analysis to assess the effect of *FLG* knockdown in an epidermal organoid has also shown features of inflammation and stress protease activity ^19^. However, *in vitro* studies have not shown consistent histological or functional effects of filaggrin deficiency in the various different skin organoid models published to date ^20^ and the multiple mechanisms by which filaggrin deficiency contributes to atopic disease remain incompletely understood ^5^.

We have optimised a skin organoid model, with dermal and epidermal compartments cultured using donor-matched primary cells, for functional assessments and global mass spectrometry proteomic analysis. The work aims to investigate in more detail the effect of *FLG* siRNA-mediated knockdown on one cell type - the keratinocyte - to increase understanding of the filaggrin-deficient phenotype and to further define molecular mechanisms predisposing to atopic skin inflammation.

## Methods

### Source of primary human cells

Primary keratinocytes and primary fibroblasts were isolated from human skin tissue samples obtained, with written informed consent and Ethical Committee approval (East of Scotland Research Ethics Service reference 17/ES/0130 renewal 12/ES/0083) under governance of the Tayside Biorepository. Surgical surplus samples of clinically normal skin from four adult donors (all females aged 29–65 years; one abdominal and three breast skin reductions) were used for the organoid cultures. Similar samples (n=5) were used for independent biological replicates to test for reproducibility of the functional effects of *FLG* knockdown.

### Organoid culture methods

Primary keratinocytes and dermal fibroblasts were isolated from human skin by sequential trypsin EDTA and collagenase D digestion ^21^. Using our previously reported methods ^12^, the keratinocytes were co-cultured with mitomycin C inactivated 3T3 feeder cells in RM media (3:1 DMEM : Hams F12, 10% FCS, 0.4µg/ml hydrocortisone, 5µg/ml insulin, 10ng/ml EGF, 5µg/ml transferrin, 8.4ng/ml cholera toxin and 13ng/ml liothyronine) (Sigma Aldrich, Gillingham, Dorset, UK) ^22^. Epidermal growth factor (EGF) was omitted for the first day of culture. The fibroblasts were cultured in DMEM supplemented with 10% FCS under standard conditions.

Fibrin gel dermal equivalents ^12^ were prepared using 0.5ml fibrinogen (35 mg/ml in NaCl) (Sigma Aldrich, Gillingham, Dorset, UK) and 0.5ml thrombin (3U/ml in 2 mM CaCl _2_ / 1.1% NaCl) (Sigma Aldrich, Gillingham, Dorset, UK) combined on ice, with 200,000 fibroblasts and aprotinin (0.1 U/ml) (Sigma Aldrich, Gillingham, Dorset, UK) then transferred to a 12-well plate. After 30 minutes incubation at 37°C, the gels were covered in medium (DMEM, 10% FCS, 0.1 U/ml Aprotinin) and cultured overnight (day 1). On day 2, the medium was replaced with RM excluding EGF, 0.1U/ml aprotinin and 2 × 10 ^6^ suspended keratinocytes. Culture medium was refreshed daily on days 3 and 4 using RM containing 0.1ng/ml EGF and 0.1U/ml aprotinin. On day 5 the gels were carefully removed from wells and lifted onto custom-made steel grids lined with nylon gauze (Millipore, Livingston, Scotland, UK). RM medium supplemented with 0.1ng/ml EGF and 0.1U/ml aprotinin was added up to the base of the dermal equivalent so that the epidermis remained at the air-liquid interface. Medium was refreshed on alternate days sand the cultures were used for analysis up to day 12 (representing three, five and seven days after lifting to the air-liquid interface).

**Figure 1.**
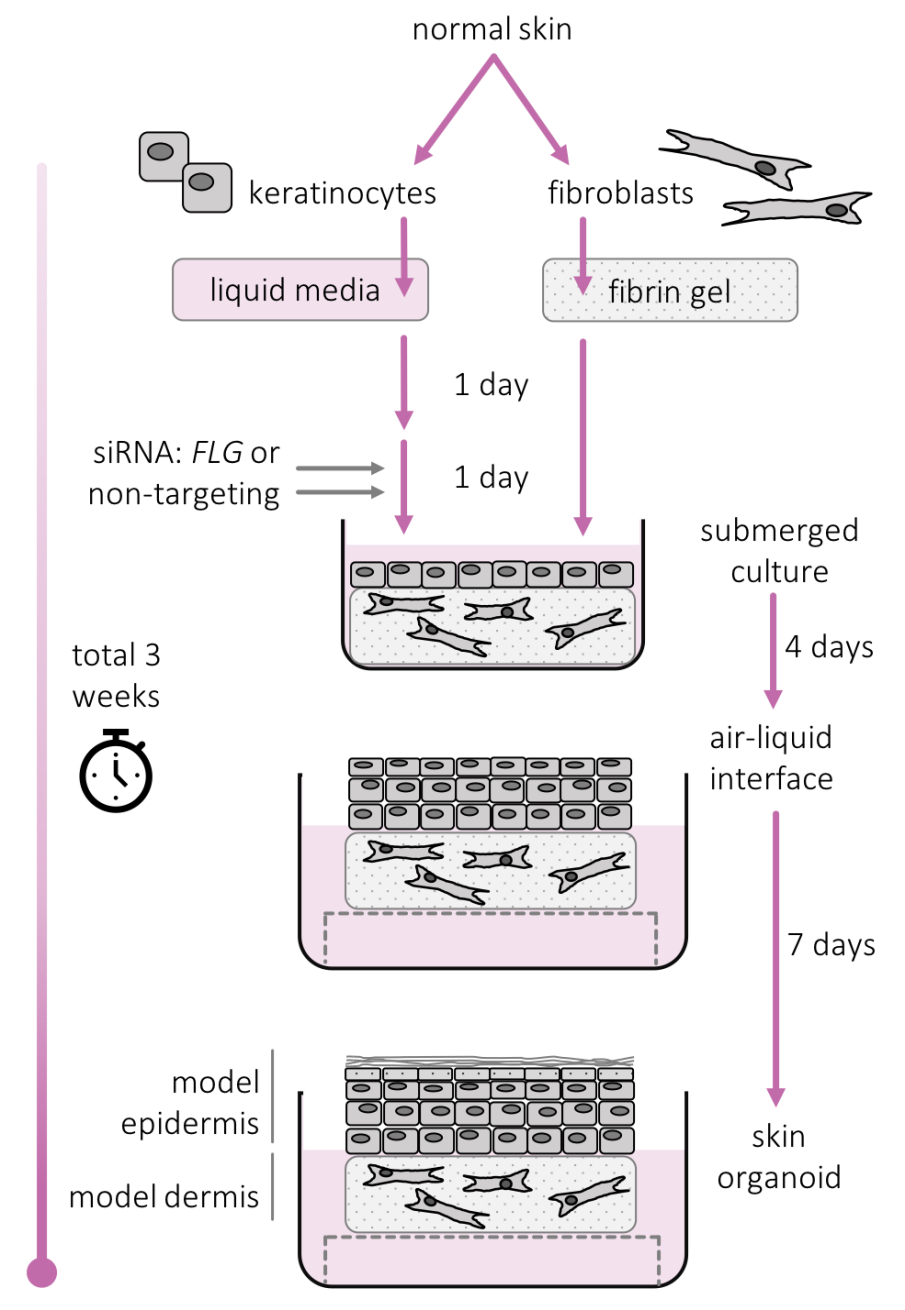
Diagrammatic summary of skin organoid culture. Dermal and epidermal equivalents produced using our published methodology optimised for skin barrier assessment ^12^.

A diagrammatic summary of the process of skin organoid culture is shown in Figure 1.

Epidermis was separated from the fibrin gel using hypertonic saline-induced split (4 hours, 1M NaCl, 4°C) to obtain a keratinocyte-only tissue sample for biochemical analyses.

### *FLG* genotyping

DNA was extracted from donor skin samples using standard techniques and genotyping was performed for the four most prevalent loss-of-function mutations in *FLG* in this white European population (R501X, 2282del4, R2447X and S3247X) ^23^ using validated KASP ^TM^ technology (LGC Genomics, Hoddesdon, Hertfordshire, UK), as previously reported ^4, 24^. Briefly, KASP ^TM^ SNP genotyping consists of two competitive, allele-specific forward primers and one common reverse primer. Each forward primer incorporates an additional tail sequence that corresponds with one of two universal FRET (fluorescent resonance energy transfer) cassettes (FAM and HEX). ROX™ passive reference dye, Taq polymerase, free nucleotides and MgCl2 in an optimised buffer solution make up the reaction master mix.

### siRNA mediated knockdown

Keratinocytes were reverse transfected immediately prior to inclusion in the organoid cultures using RNAiMax transfection reagent (Life Technologies, Carlsbad, California, USA) according to manufacturer’s instructions. Briefly, siRNA complexes were formed in OPTIMEM medium (20uM siRNA, 5µl RNAiMAX) and following 20 minutes incubation, combined with 2 × 10 ^6 ^suspended normal human keratinocytes and transferred to the pre-prepared dermal substrate. A pool of four siRNA duplexes was used [FLG: LQ-021718-00-0002, Control: ON-TARGETplus non-targeting siRNA #4 D.001810-04-20] (Dharmacon, Lafayette, Colorado, USA).

### Quantitative PCR

RNA was extracted from organoid epidermis using the Direct-zol RNA kit (R2071, Zymo Research, Irvine, California, USA) following homogenisation in RNA-Bee (CS-104B, Amsbio, Abingdon, Oxfordhire, UK) using the TissueLyser LT (Qiagen, Manchester, UK) 5 mins at 50 Hz. cDNA was prepared from 1μg total RNA using random priming (2.5µM final) (Integrated DNA Technologies, Coralville, Iowa, USA) in combination with Moloney murine leukemia virus (MMLV) enzyme (100 units) and buffer systems (28025013, Life Technologies, Carlsbad, California, USA). qPCR reactions were performed using exon spanning probe-based assays ( *FLG*: HS.PT.58.24292320FAM, *EF1A*: HS.PT.58.24345862FAM) [Integrated DNA Technologies, California, USA]) with TaqMan gene expression Mastermix (4369016, Life Technologies, Carlsbad, California, USA). Reactions were prepared and run using the Qiagility robot in combination with the Rotor-Gene Q (Qiagen, Manchester, UK). *EF1A* was used as a reference gene. PCR cycling conditions were: 95°C for 10 mins, [95°C for 15 seconds, 60°C for 60 seconds] × 40 cycles. Fold changes were derived via the 2(-Delta Delta C[T]) method.

### Western blotting

Organoid epidermis was homogenized in RIPA buffer (Cell Signalling Technologies, London, UK) using the TissueLyser LT (Qiagen, Manchester, UK) 5 mins at 50 Hz. Protein lysates were normalized by Pierce BCA assay (23225, Thermo Fisher, Waltham, Massachussets, USA) and resolved under reducing conditions using the NuPage® gel electrophoresis system (Life Technologies, Carlsbad, California, USA). Primary antibodies (monoclonal mouse filaggrin [RRID: AB_1122916, catalogue # sc66192, Santa Cruz Biotechnology, Texas, USA] and monoclonal mouse GAPDH [RRID: AB_2756824, catalogue # 97166, Cell Signalling Technologies, Boston, USA]) were prepared in 5% bovine serum albumin (BSA) and incubated overnight at 4°C with agitation. Immunoblots were developed using either goat anti rabbit (P0448, RRID: AB_2617138) or goat anti mouse (P0447, RRID: AB_2617137) peroxidase conjugated secondary antibodies (DAKO, Glostrup, Denmark, 1:5000) in combination with chemiluminescent HRP substrate (Immobilon, Millipore, Billerica, Massachussets, USA) onto photographic film. Densitometry was performed using Fuji ImageJ (May 2017).

### Structural assessments

(i) Histology

Formalin-fixed, paraffin embedded full thickness organoids were sectioned and stained using haematoxylin and eosin and imaged using Visiscope LT384P (VWR International, Lutterworth, UK).

(ii) Transmission electron microscopy (TEM)

Organoid skin samples were fixed in 4% paraformaldehyde, 2.5% gluteraldehyde in 0.1M sodium cacodylate buffer (pH 7.2) for one hour then cut into small pieces, washed in buffer and post-fixed in 1% osmium tetroxide in cacodylate buffer for one hour or 1% ruthenium tetroxide in cacodylate buffer for one hour. The pieces were dehydrated through alcohol series, into propylene oxide and embedded in Durcupan resin. Ultrathin sections were stained with 3% aqueous uranyl acetate and Reynold’s lead citrate and examined on a JEOL 1200EX electron microscope. TEM images were collected on a SIS Megaview III camera.

### Functional assessments

(i) Trans-epidermal water loss (TEWL)

Organoid cultures were equilibrated at room temperature and atmospheric conditions for 30 minutes before TEWL was measured at two locations on the epidermal surface using an Aquaflux AF200 instrument (Biox Systems Ltd, London, UK) with a closed chamber and a custom (5mm diameter) probe head. TEWL measurements were taken every second for a minimum of 60 seconds until a stable reading, as determined by the software, was obtained. TEWL directly measures water evaporation from the skin surface but it may be considered as a measurement of ‘inside-outside’ barrier function ^25^.

(ii) Capacitance

To inform the interpretation of TEWL, we measured electrical capacitance; this is directly proportional to water content of the uppermost 10–20µm of tissue. Organoid cultures were equilibrated at room temperature and atmospheric conditions for 30 minutes prior to measurement of epidermal surface capacitance, using a Corneometer ^TM^ and multiprobe adapter (CM825 and MPA2, Courage and Khazaka, Cologne, Germany). Three measurements were recorded from each organoid and the mean was calculated.

(iii) Fluorescent dye penetration

50μl of 1mM lucifer yellow dye (Sigma Aldrich) was added to the epidermal surface of the organoid and incubated at 37°C for 4 hours. Metal cloning rings were used to control uniform dosing on the epidermal surface. The lucifer yellow was removed and the organoids washed in PBS before formalin fix-paraffin embedding under standard conditions. 4µM sections were deparaffinized, counterstained with DAPI (1μg/ml for 10 mins) (Life Technologies, Carlsbad, California, USA) and imaged by confocal Zeiss LSM710 microscope. Quantification of dye penetration in the upper dermis (average intensity in upper 40μm) was performed using Zeiss Zen Blue lite version 2.3 software and compared using paired t-tests. Dye penetration represents a measurement of the ‘outside-inside’ barrier function.

### Mass spectrometry proteomic analysis

Frozen organoid epidermal samples were ground on dry ice, solubilized in 200μl of Cellular and Organelle Membrane Solubilizing Reagent (C0356-4BTL, also called Protein Extraction Reagent Type 4, Sigma-Aldrich, Gillingham, Dorset, UK), 7.0 M urea, 2.0 M thiourea, 40 mM Trizma® base (Sigma Aldrich, Gillingham, Dorset, UK) and 1.0% C7BzO, pH 10.4 buffer with protease inhibitors. The lysate was acetone precipitated for 1 hour at -20°C and spun for 20 mins at 2°C at 15,000 rpm. The pellet was re-suspended in 200μl of 50mM ammonium bicarbonate. 100μl was taken for in-solution digest. 22μl of dithiothreitol was added and samples heated to 50°C for 15mins. 24μl of iodoacetamide was added and the samples incubated at 22°C for 30 mins. 1μl of RapiGest ^TM^ (186001860, Waters, Herts, UK) was added, followed by 2.5μl of trypsin, for a 12-hour digest. A further 1μl trypsin was added for an additional 4-hour digest. The peptide samples were fractionated into 24 fractions with high pH Reversed Phase C18 chromatography, run on a Q Exactive Classic or Plus for 160 mins and the top 15 ions selected for sequencing. Mass spectrometry resolution was 70,000 and tandem mass spectrometry (MS/MS) resolution 17,500. Data were processed using MaxQuant (v1.6.0.13) and Human UniProt Database (Dec 2017) with a protein and peptide false discovery rate of 0.01.

Proteins identified as contaminants (trypsin and other lab originating proteins) and contained within the MaxQuant database were removed. To account for the possibility of keratin as a contaminant, we analysed the blank runs for keratin peptide intensity and subtracted this from the measured keratin intensities. A reversed database was used to identify false positives. The peptide and protein false discovery rate was set at 1%, and these were removed from further analysis.

Total intensities of the samples were normalized to total protein abundance, with adjustment to the lowest total protein yield. All samples were run in quadruplicate and reproducibility was assessed by Pearson correlation.

### Mass spectrometry lipidomic analysis

Organoid samples were sonicated in phosphate buffered saline (pH7.4) and then extracted according to the method of Folch *et al.* ^26^. The samples were analysed by liquid chromatography-mass spectrometry (LC-MS) on a Thermo Exactive Orbitrap mass spectrometer (Thermo Scientific, Hemel Hempsted, UK) coupled to a Thermo Accela 1250 ultra high pressure liquid chromatography (UHPLC) system. Samples were injected on to C18 column (Thermo Hypersil Gold, 2.1 mm x 100 mm, 1.9 μm) and separated using a water/acetonitrile/isopropanol gradient ^27^. Ceramide 17:0 (Avanti Polar Lipids, Alabaster, AL, USA) was included as an internal standard. Ion signals corresponding to individual ceramide molecular species were extracted from raw LC-MS data sets. Concentration of ceramide was expressed as pmol/mg after normalisation to mg of wet weight tissue.

### Data analysis of qPCR, immunoblotting and functional assessments

Measurements were compared between non-targeting control-treated organoid models and *FLG*-siRNA-treated models, using paired t-test.

Histological sections of 10 replicate skin organoid experiments were measured using a standardized technique to minimize bias, as follows: three points were measured on each slide, at the right side, middle and left of each sample, across viable cell layers and stratum corneum; measurements were generated using Fuji ImageJ (May 2017) and the mean of the three measurements calculated.

### Proteomic data analysis

The total intensities of the samples were normalised to total protein abundance, with adjustment to the lowest total protein yield. Log2 ratio of protein expression (log2 FLG knockdown – log2 NT control) was visualised as a volcano plot, using GraphPad Prism (version 5) for human proteins detected in (three or all four) of the replicate experiments. P values were calculated by paired t-test applied to log 10 transformed data. Because of the burden of multiple testing in this large dataset, rather than defining arbitrary thresholds for ‘statistical significance’, our analyses focussed on changes that showed consistency across the biological replicate experiments. Approximately 1000 of the >8000 proteins were selected as showing consistently increased or decreased abundance, defined as follows:

Proteins were considered to be reduced in abundance if they showed a reduction in all four biological replicate experiments comparing *FLG-*knockdown organoid samples to matched non-targeting controls and ratio ≤0.5 in three or four out of four of the replicates and reduced to undetectable levels of protein in a maximum of two replicates. Proteins were considered to be increased in abundance if they showed an increase in all four biological replicate experiments comparing *FLG-*knockdown organoid samples to matched controls treated with non-targeting controls and ratio ≥1.2 in three or four out of four of the replicates. These magnitudes of change were used to define roughly equal proportions of up- and down-regulated proteins for pathway analysis. The groups were then assessed using gene ontology and network analysis in STRING, version 11 ^28^ and pathway prediction using the Reactome Knowledgebase, version 68 ^29^. This analysis, based on consistency of findings across multiple biological replicate experiments was used in preference to a defined threshold of statistical significance, with the aim to identify proteins and pathways of importance in the context of a common complex but heterogenous trait.

### Lipidomic data analysis

The ratio of ceramide and omega-hydroxy ceramide species were compared between the *FLG* knockdown organoids and matched control samples in five biological replicate experiments.

## Results

### All samples are wild-type for the prevalent *FLG* null mutations and filaggrin shows knockdown at mRNA and protein level

Genotyping of DNA from tissue samples from the four skin donors did not detect any of the prevalent *FLG* null mutations ^30^; this does not, however, exclude the possibility of rare (<0.01 allele frequency) null mutations.

**Figure 2.**
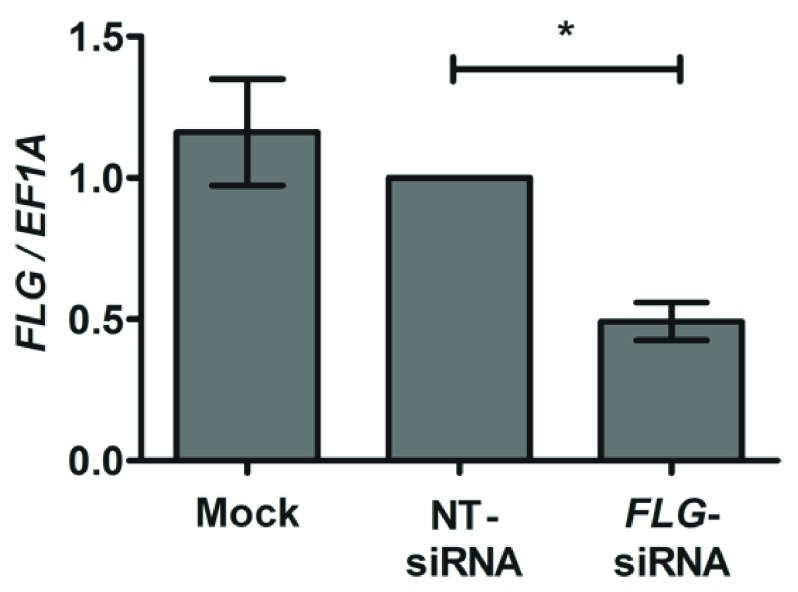
qPCR analysis of epidermis from skin organoids with and without *FLG* siRNA-mediated knockdown. Probes and primers are listed in Methods; n=8 biological replicates; error bars show SEM; *paired t-test p<0.05.

**Figure 3.**
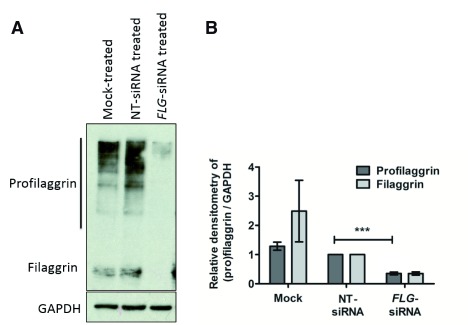
Quantification of filaggrin in organoid epidermis. ( **A**) Representative western blot. ( **B**) Approximated quantification by densitometry. NT, non-targeting; n=7; ***p<0.0005 compared to non-targeting control.

*FLG* knockdown in the skin organoids persisted to day 10 after siRNA transfection, as demonstrated by qPCR ( Figure 2) and western blotting ( Figure 3). We note the greater magnitude of knockdown quantified at mRNA than protein level in the organoid epidermis 10-days after transfection. This is likely to reflect the inherent differences in mRNA and protein stabilities: FLG mRNA has a half-life of ~2.2 hours ^31^ whereas the half-lives of profilaggrin and filaggrin are approximately 6 and 24 hours respectively ^32^.

### *FLG* knockdown results in morphological changes in the epidermis

**Figure 4.**
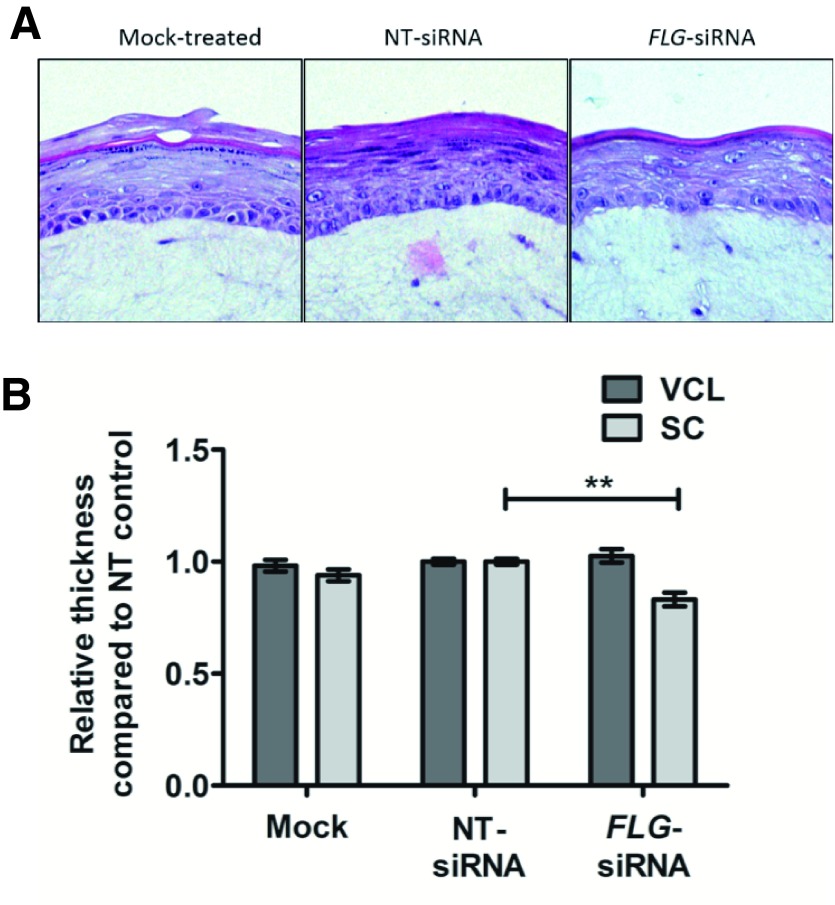
Structural changes in organoid models. ( **A**) Histological examination (representative images from 8 biological replicates). NT, non-targeting. ( **B**) Measurement of relative thickness of epidermal layers: VCL, viable cell layer and SC, stratum corneum; n=8; compared using paired t-test; **p<0.005.

Histological examination confirmed a reduction of the granular layer, in keeping with a reduction in profilaggrin ( Figure 4A). There was also a slight reduction in thickness of the stratum corneum (ratio 0.83 +/- 0.05 (mean +/- SEM), but no difference in thickness of the keratinocyte cell layers ( Figure 4B).

**Figure 5.**
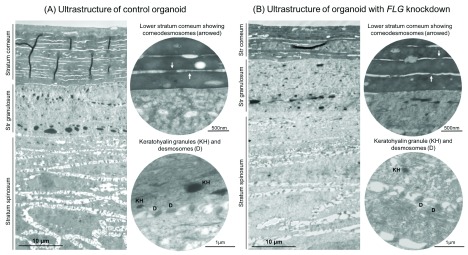
Ultrastructural features of control and *FLG* knockdown organoids. ( **A**) Control organoid sample, showing elements of normal human skin structure including stratified differentiation. ( **B**) Organoid with *FLG* siRNA-mediated knockdown, showing thinner stratum corneum, reduced prominence if the corneodesmosomes and desmosomes, and smaller keratohyalin granules (organelles containing profilaggrin).

Transmission electron microscopy (TEM) of the skin organoid model shows an effective recapitulation of the physiological layers of keratinocyte differentiation within skin, including a mature, multi-layered stratum corneum with corneodesmosomes and a granular layer with keratohyalin granules and desmosomes ( Figure 5A). TEM of the *FLG-*knockdown organoid shows a similarly mature epidermis, but the stratum corneum has fewer layers and the granular layer contains keratohyalin granules that are generally smaller and less numerous than in the control organoid ( Figure 5B).

### *FLG* knockdown shows some evidence of a barrier defect in the immature organoids but no functional effect on skin barrier in the mature organoid model

**Figure 6.**
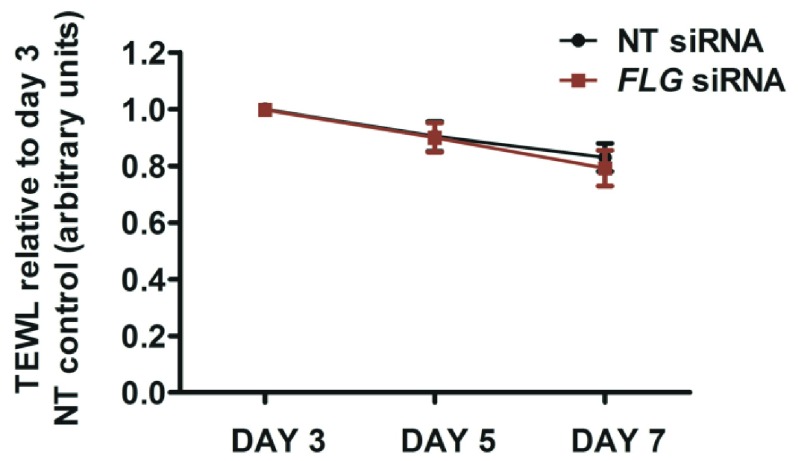
Transepidermal water loss (TEWL) measurements. TEWL measured under standard conditions at days 3, 5 and 7 after the organoid is lifted to the air-liquid interface, using an Aquaflux AF200 instrument (Biox Systems Ltd, London, UK) with a 5mm diameter probe head. NT, non-targeting; N=5; error bars show SEM.

**Figure 7.**
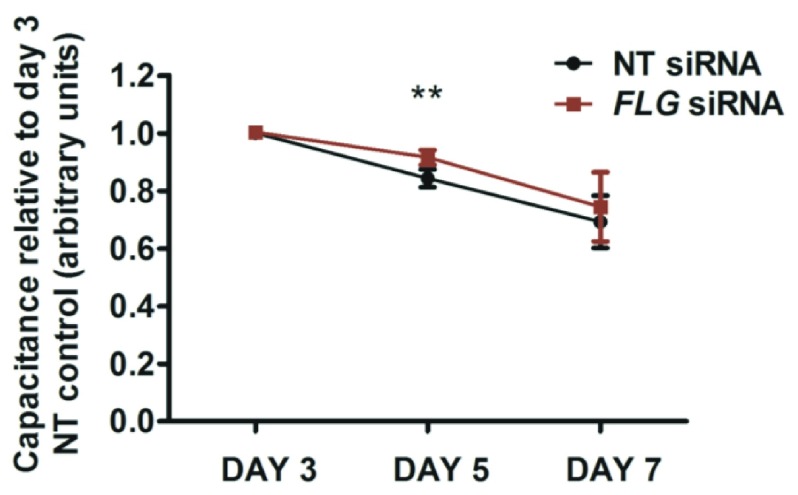
Epidermal capacitance measurements. Electrical capacitance, directly proportional to water content of the uppermost 10–20µm of tissue, was measured under standard conditions at days 3, 5 and 7 after the organoid is lifted to the air-liquid interface, using a Corneometer ^TM^ (Courage and Khazaka, Cologne, Germany); the mean of three measurements is recorded. NT, non-targeting; N=5; error bars show SEM; paired t test **p<0.005.

**Figure 8.**
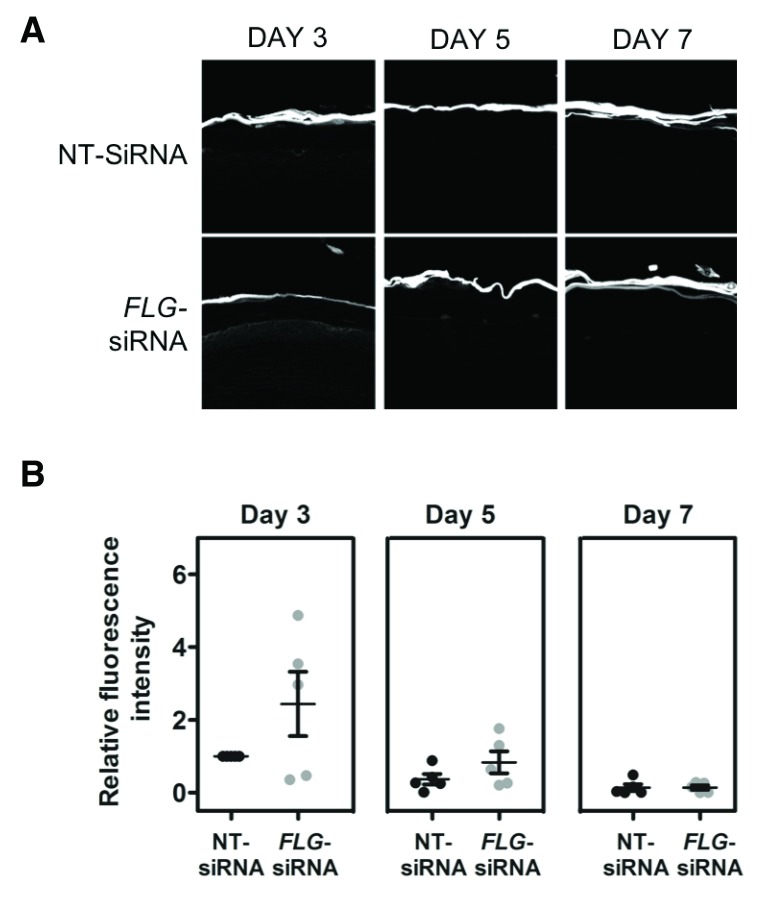
Lucifer yellow dye penetration of organoid samples. The hydrophilic lucifer yellow dye (Sigma Aldrich) was added to the epidermal surface of the organoid and incubated at 37°C for 4 hours before sectioning for microscopic examination. ( **A**) Representative image from three of the five biological replicate experiments showing delayed maturation of barrier function. ( **B**) Dot-plot of densitometry data from all five biological replicates. NT, non-targeting.

The organoid model described here shows progressive development of skin barrier function after lifting to the air-liquid interface: there is a progressive reduction in TEWL ( Figure 6), progressive reduction in capacitance ( Figure 7) and the hydrophilic dye, lucifer yellow, is excluded from the epidermis by day 7 in both the control and *FLG*-knockdown organoids ( Figure 8).

Results from five biological replicate experiments showed there was no difference in the mean TEWL ( Figure 6), or mean capacitance measurements ( Figure 7) between the mature organoid models with *FLG* knockdown and donor-matched non-targeting siRNA-treated controls. Capacitance was increased in the immature *FLG-*knockdown model (day 5 after air-exposure, Figure 7) and lucifer yellow dye was not fully excluded by the stratum corneum in some of the the *FLG-*knockdown replicate experiments until day 7 ( Figure 8). However, this delay in maturation of the ‘outside-to-inside’ barrier function was not consistently demonstrated in all biological replicates.

### Mass spectrometry proteomic analysis identifies over 8000 protein species per sample

**Figure 9.**
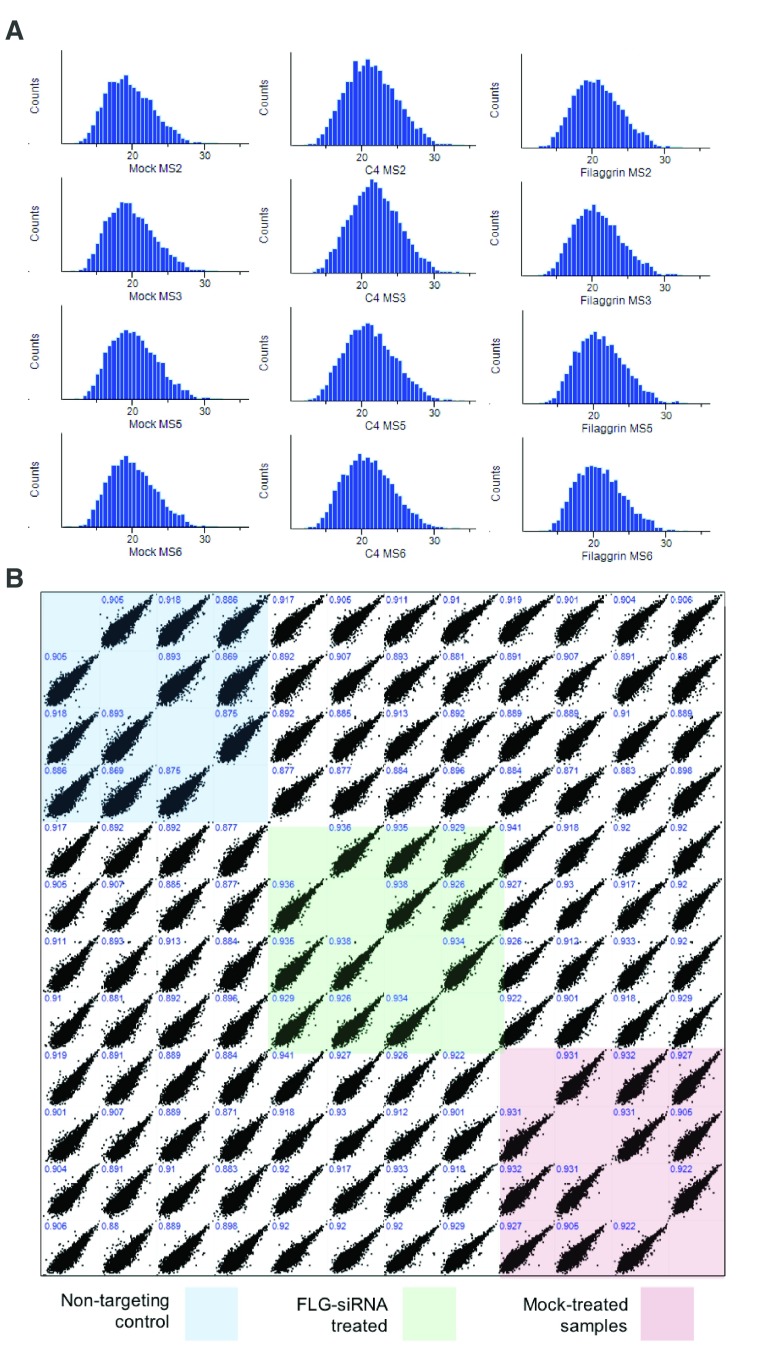
Mass spectrometry proteomic analysis quality control assessments. ( **A**) Histograms showing the distribution of proteins quantified in each experiment. Bins on the x-axis contain the log2 intensity-based absolute quantification (iBAQ, sum of intensities of tryptic peptides for each protein divided by number of theoretically observable peptides); normalised counts of the proteins in each bin are shown on the y-axis; MS2, MS3, MS5 and MS6 are biological replicate experiments from different human donors; mock, untreated control organotypic; C4, non-targeting control treated organotypic; Filaggrin, FLG si-RNA treated organotypic. ( **B**) Correlation of protein abundance from replicate analyses. x and y axes show iBAQ intensities of samples mapped against replicate analysis; biological replicates are highly correlated (Pearson correlation coefficient ≥0.86).

**Figure 10.**
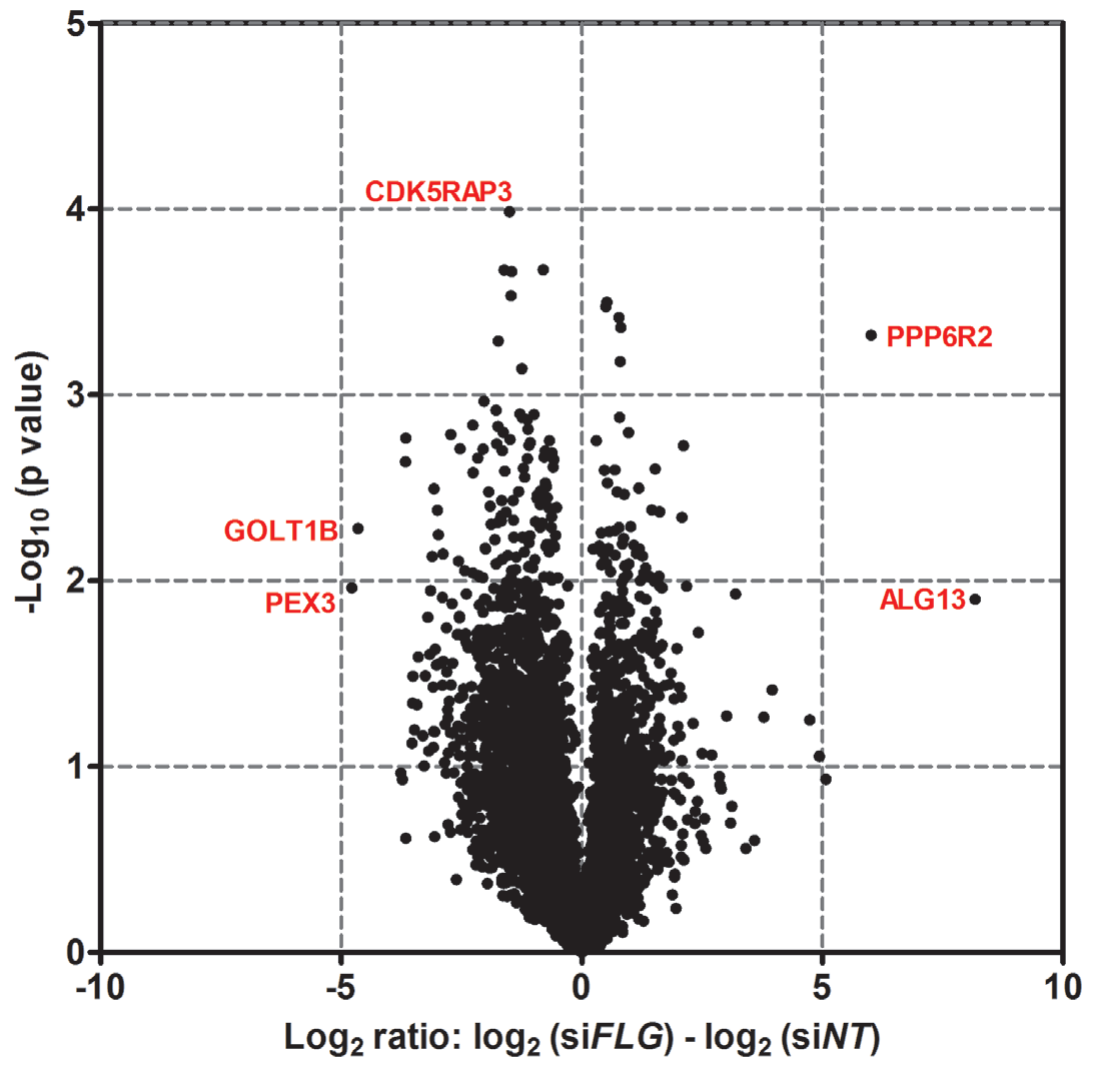
Volcano plot to visualise changes in protein expression between control and *FLG* siRNA-treated organoids. Adjusted protein intensity data were filtered to include only human proteins detected in three or four replicate experiments. The log2 ratio (log2 FLG knockdown – log2 NT control) for each donor was calculated and averaged; p-values from paired t-test were derived from log10 transformed data and are unadjusted. Selected data-points with more extreme values are labelled using the abbreviated protein name. NT, non-targeting.

After removal of contaminants and false positive results, >8000 proteins were identified in each epidermal sample. The proteomic data have been deposited to the ProteomeXchange Consortium via the PRIDE ^33^ partner repository with dataset identifier PXD014875. Quality control analysis confirmed that similar distributions of proteins were identified in each experiment ( Figure 9A); protein extracts were analysed in quadruplicate to test for technical reproducibility and the Pearson correlations were 0.869-0.938 ( Figure 9B). The protein expression changes were visualised using a volcano plot ( Figure 10).

### 648 proteins show a consistent reduction in expression in skin organoids with FLG knockdown

648 human proteins showed a reduction in expression in all four of the replicates and ratio ≤0.5 in three out of four replicates. This list is available as *Underlying data*, uploaded to Figshare ^30^.

645 of the proteins are identified in STRING and this group showed significant enrichment for interactions (p<1.0e-16) computed by combining the probabilities from the different evidence channels and corrected for the probability of randomly observing an interaction ^21^. Gene ontology (GO) analysis in STRING showed evidence for functional enrichment of GO-terms including RNA processing (false discovery rate (FDR) 4.40e-11), catalytic activity (FDR 8.59e-08) and intracellular component (FDR 3.88e-26). A list of the 10 most significant GO analysis results in each of the GO domains is shown in Table 1 (see *Underlying data*) ^34^.

### Pathway analysis of down-regulated proteins identifies themes including RNA metabolism, adaptive immunity and axon guidance

Reactome detected 39 pathways in this set of proteins (Table 2, *Underlying data*) ^35^. Descriptions include: metabolism of RNA (including 67 out of the 652 genes in this pathway, FDR 3.36e-12); class I MHC mediated antigen processing and presentation (30/365 genes in pathway, FDR 0.0031); and axon guidance (34/541 genes, FDR 0.0207).

### Down-regulated proteins indicate molecular mechanisms of relevance to atopic eczema

Manual searching of the list of down-regulated proteins identified several of specific interest based on *a priori* knowledge of eczema pathomechanisms. These highlight several putative therapeutic targets: the aryl hydrocarbon receptor plays a role in signalling pathways for skin barrier repair ^22^ and has been proposed as a therapeutic target for the treatment of skin inflammation ^36^; caspase 14 plays a role in filaggrin processing ^37^ and is required for epidermal protection against water loss and UVB-induced damage ^38^; dermokine is a soluble regulator of keratinocyte differentiation ^39^; sequence similarity to a murine protein (UniProtKB: Q8R1R3) indicates that StAR-related lipid transfer protein 7 may play a role in protecting mucosal tissues from exaggerated allergic responses; a reduction in the cytokine TGF-beta 1 may predispose to atopic eczema ^40^; and loss-of-function mutations in *CYP4F22* cause lamellar ichthyosis ^41^, suggesting that a reduction in CYP4F22 protein expression may contribute to the ichthyotic phenotype observed in ichthyosis vulgaris and atopic skin.

### 376 proteins show a consistent increase in expression in organoids with FLG knockdown

376 human proteins showed an increase in expression in all four of the replicates and ratio ≥1.2 in three or four out of the four replicates. This list is available as *Underlying data*, uploaded to Figshare ^30^.

374 proteins are identified in STRING and the group showed significant enrichment for interactions (p<1.0e-16). GO analysis revealed enrichment of terms including translation (FDR 4.12e-19), RNA binding (FDR 9.77e-12) and cytoplasmic part (FDR 9.64-36). A list of the 10 most significant GO analysis results in each of the GO domains is shown in Table 3 (see *Underlying data*) ^42^.

### Pathway analysis of up-regulated proteins identifies themes including translation, innate and adaptive immunity and axon guidance

Reactome detected 113 pathways in this set of proteins with FDR p<0.05 (Table 4, *Underlying data*) ^43^. Descriptions include: Translation (including 44 out of the 288 genes in this pathway, FDR 1.17e-21); innate immune system (47/1012 genes in pathway, FDR 9.90e-7); cytokine signalling in immune system (32/654 genes in pathway, FDR 5.16e-05); and axon guidance (40/541, FDR 4.69e-11).

### Up-regulated proteins indicate molecular mechanisms of relevance to atopic eczema

Manual searching of the list of up-regulated proteins identified several of relevance to atopic eczema, including: STAT1, an intracellular signalling molecule that shows increased expression in atopic eczema, and as part of the JAK-STAT pathway is targeted by several traditional herbal remedies ^44^ as well as novel small molecule therapies ^45^; carbonic anhydrase 2, which is increased in skin in a variety of forms of eczema ^46^; and FK binding protein, which binds to tacrolimus, an established topical treatment for atopic eczema.

The type I keratins 10, 16 and 17 each show increased expression in the *FLG*-siRNA-treated organoid models with mean ratio 1.7, 1.3 and 1.3, respectively. Keratin 10 is an intermediate filament protein of structural importance in the skin; keratin 16 is an epidermis-specific keratin that plays a role in innate immunity in response to skin barrier breach ^47^; keratin 17 is expressed in epidermal appendages, including the hair follicle.

Finally, it is noteworthy that GAPDH, which is often considered to be a stable ‘housekeeping’ gene, is in the dataset of consistently up-regulated proteins. However, in the context of this work, filaggrin knockdown is clearly apparent at a greater magnitude than the more subtle change in GAPDH ( Figure 3).

### Epidermal ceramide content shows no significant difference in FLG-knockdown organoids compared with controls

**Figure 11.**
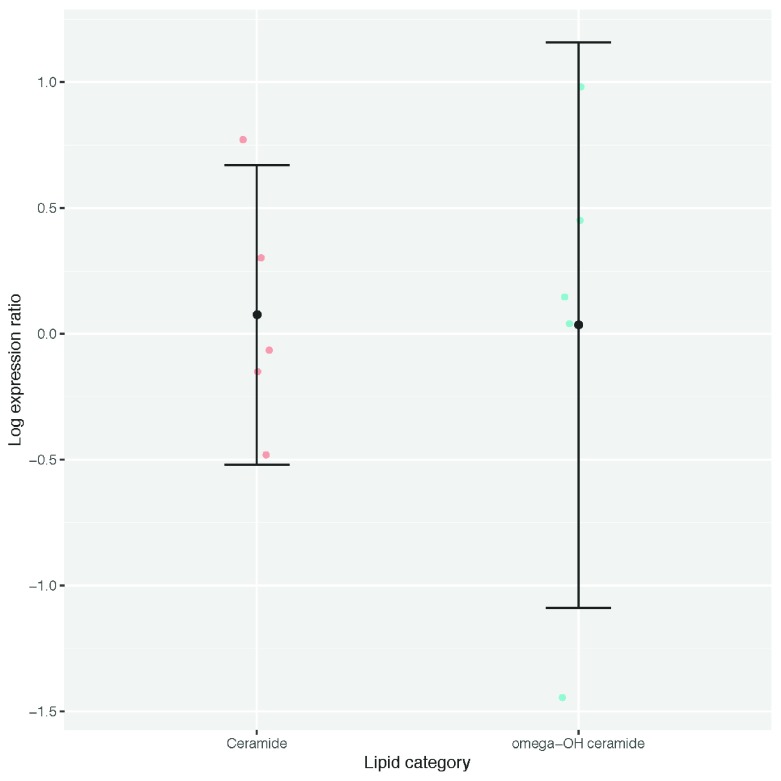
Ratio of ceramides and omega-hydroxy ceramides in organoid epidermis. Dots represent values for five individual experiments; bars show 95% confidence interval.

Ceramide and omega-hydroxy ceramide species in five biological replicate experiments showed no consistent difference between the *FLG* knockdown organoids and matched control samples ( Figure 11).

## Discussion

The functional and biochemical analyses of a filaggrin-deficient skin model have provided findings that are consistent with our and others’ previous analyses *in vitro* ^19^ and *in vivo* ^48^. We have included both epidermal and dermal compartments in this model (to allow for dermal-epidermal cross-talk ^49, 50^) and our more detailed proteomic analysis has revealed additional evidence of molecular mechanisms with relevance to atopic skin. These datasets are shared as a resource for the research community.

Analysis of the immature organoid identified a delay in epidermal maturation following *FLG* knockdown, but the lack of a replicable functional effect in our model, when mature, reflects previous reports in which epidermal equivalents created from *FLG*-null keratinocytes do not show impairment in barrier function ^20^. This is in keeping with the observation that *FLG*-null mutations are not fully penetrant with respect to ichthyosis vulgaris or atopic eczema ^16, 51^.

Because of the nature of our data, generated using biological replicate samples, and the focus on modelling a common complex trait with considerable inter-individual variation, we elected to use the criterion of consistency across the biological replicates to define proteins of interest, followed by network and pathway analyses. We recognise that other, more complex data analytical approaches may be applied, and/or more focussed interrogation of specific pathways; we share the proteomics data and welcome such further analysis.

Our analysis has highlighted aspects of keratinocyte-immune signalling. This is an important component of the immunological barrier that may be overlooked in tissue with multiple different cell types. The predicted pathways entitled ‘innate immunity’ and ‘adaptive immunity’ included proteins showing both increased and decreased expression (Table 2 and Table 4, *Underlying data*) ^35, 43^. This immune dysregulation is likely to contribute to the predisposition to infection and inflammation observed in filaggrin-deficient skin *in vivo*: filaggrin-deficient patients show increased viral infection of the skin ^52^ and increased incidence of irritant ^53, 54^ and allergic contact dermatitis ^55^. The regulation of innate and adaptive immune pathways within keratinocytes (in addition to cells of the haematopoietic lineage) may therefore represent a worthwhile target for future therapeutic interventions.

The lack of effect of *FLG* knockdown on the ceramide content in the model epidermis is also consistent with previously reported findings *in vitro ^56, 57^* and *in vivo* ^58^. We did not analyse in detail the other epidermal lipids and therefore we cannot comment on whether there were relevant changes. Differences in lamellar body structure ^59^ have been reported in *FLG-*mutant skin biopsies but this was not clearly observed in the organoid model. However, ultrastructural examination of the model stratum corneum did reveal abnormalities in the lamellar bilayer structure and less prominent corneodesmosomes ( Figure 5), as previously reported *in vivo* ^59^. It is also noteworthy that pathway analysis in Reactome identified the terms ‘metabolism of lipids’ and ‘phospholipid metabolism’ from the group of consistently down-regulated proteins (Table 4, *Underlying data*) ^43^.

The most significant themes identified in the groups of proteins showing increased expression upon *FLG* knockdown relate to RNA binding and translation; this is in contrast to RNA metabolism and RNA processing, which are demonstrated in the proteins showing decreased expression. The functional significance of this increase in transcriptional and translational activity cannot be determined from the gene ontology and pathway analysis. We may hypothesise that it reflects the increased ‘stress’ response, as we have previously observed in *FLG* genotype-stratified transcriptome analysis of atopic skin biopsies ^18^ and/or the activation of innate and adaptive immune mechanisms.

It is tempting to speculate that the increased expression of keratins 10 and 16 may contribute to palmar hyperlinearity and keratin 17 may contribute to keratosis pilaris. These are characteristic features of ichthyosis vulgaris but their pathogenesis has not yet been explained as a direct result of filaggrin haploinsufficiency ^16, 60^.

Several of the proteins showing reduced expression relate to aspects of differentiation, including filaggrin itself, and caspase 14 which plays a role in the terminal degradation of filaggrin ^37^. The aryl hydrocarbon receptor (AhR) is a ligand-activated transcription factor that responds to multiple different environmental and metabolic stimuli to control transcriptional pathways of relevance to atopic eczema, including immunity and differentiation ^61^. Activation of AhR reduces skin inflammation ^62^ and enhances barrier repair ^63^; therefore, a reduction in AhR expression in the *FLG* knockdown organoid would be anticipated to increase the eczema diathesis by opposing these effects. AKT1 activity is known to be reduced in atopic skin, leading to an alteration in protease expression and reduced filaggrin expression and processing ^64^. This mechanism may further exacerbate the filaggrin deficiency in our *FLG* knockdown organoid. Dermokine acts as a soluble regulator of keratinocyte differentiation and mice deficient in the epidermal dermokines (beta and gamma isoforms) show defective cornification ^65^. Transforming growth factor (TGF) beta-1 is a multifunctional protein: it regulates the growth and differentiation of multiple cell types, including keratinocytes and it can promote either Th17 or T-regulatory cell lineage differentiation in a concentration-dependent manner; it appears to have an immunosuppressive effect in atopic disease since a genetically-determined lower production of TGF-beta-1 is associated with increased risk of atopic dermatitis ^40^.

The Reactome term ‘axon guidance’ was detected in proteins showing increased and decreased expression. It has long been recognised that there is an increase in the density of sensory neurons in eczematous skin ^66^ and nerve growth factor expression is upregulated in the keratinocytes of patients with atopic eczema ^67^. Therefore, dysregulation of axon guidance may be of relevance to the pruritus that is so characteristic of atopic eczema and contributes substantially to the morbidity of this condition ^3^. Antagonists of transient receptor potential vanilloid subfamily member 1 (TRPV1), expressed on sensory nerves and keratinocytes, are now being investigated for the control of pruritus ^68, 69^.

Taken together, our data demonstrate that filaggrin haploinsufficiency leads to abnormalities in both structural and immune features within the keratinocyte compartment of the epidermis. Keratinocytes display immune activity, producing multiple cytokines and chemokines in addition to their roles in innate immunity ^70, 71^. Together with the evidence of dysregulation in axon guidance, these findings indicate that patients with filaggrin deficiency – either haploinsufficiency or a reduction in filaggrin expression that occurs secondary to atopic inflammation ^72^ – may derive clinical benefit from future therapies targeting keratinocyte-immune crosstalk mechanisms and neurogenic pruritus.

The skin organoid model described here represents a valuable opportunity to study genetic effects on keratinocytes in relative isolation. However, the lack of other cell types (notably T cells, B cells, dermal dendritic cells and innate lymphoid cells), the absence of complex dermal structures (including neurons and blood vessels) and skin appendages (for example hair follicles, sweat and sebaceous glands) is also a limitation in that it precludes assessment of the complex interactions that are likely to occur between different tissue compartments in skin.

In conclusion, we share these experimental results in detailed form, because of the additional insight provided by this *in vitro* analysis of filaggrin deficiency in a skin organoid model; our findings compliment and extend previous work *in vitro* and *in vivo*. Our analyses have re-emphasised the role of keratinocyte-specific mechanisms in contributing to immune and neurological as well as structural aspects of skin barrier function and offer new insight into molecular mechanisms for the ichthyosis vulgaris phenotype.

## Data availability

### Underlying data

Mass spectrometry proteomics data deposited in the ProteomeXchange Consortium via the PRIDE partner repository ^33^, Accession number PXD014875: https://identifiers.org/pride.project:PXD014875

Figshare: Proteomic analysis of *FLG* knockdown in skin organoid. https://doi.org/10.6084/m9.figshare.c.4606052.v3 ^30^.

This project contains the following underlying data:

- TEM images (zip file containing raw, unedited TEM images in .tif format)
- TEWL and corneometer readings (raw readings in .xlsx format)
- Western blots (zip file containing unedited western blot scans, plus an annotated composite image with labels in .tif format)
- Lucifer yellow dye penetration quantification.xlsx
- Lucifer yellow dye penetration (zip file containing raw, unedited microscopy images for five replicates in .czi format)
- Histology quantification (raw histology quantification data in .xlsx format)
- H&E images (raw, unedited histology images for replicates 1-11 in .bmp and .tif format)
- Filaggrin densitometry (densitometry measurements for seven replicate experiments in .xlsx format)
- FLG qPCR data (raw qPCR data in .xlsx format)
- FLG genotyping results (raw genotyping results in .xlsx format)
- Consistently down-regulated proteins in FLG kd organoids.xlsx
- Consistently up-regulated proteins in FLG kd organoids.xlsx

Figshare: Table 1. Gene Ontology (GO) analysis of proteins showing a consistent reduction in expression with *FLG* knockdown. https://doi.org/10.6084/m9.figshare.9710585.v1 ^34^.

Figshare: Table 2. Reactome pathway analysis of down-regulated proteins. https://doi.org/10.6084/m9.figshare.9710666.v1 ^35^.

Figshare: Table 3. Gene Ontology (GO) analysis of proteins showing a consistent increase in expression with *FLG* knockdown. https://doi.org/10.6084/m9.figshare.9710738.v1 ^42^.

Figshare: Table 4. Reactome pathway analysis of up-regulated proteins. https://doi.org/10.6084/m9.figshare.9710783.v1 ^43^.

Data are available under the terms of the Creative Commons Attribution 4.0 International license (CC-BY 4.0).
