# Supplementary material for: Functional and proteomic analysis of a full thickness filaggrin-deficient skin organoid model
Source: Wellcome Open Res. 2019 Nov 26;4:134. Originally published 2019 Sep 9. [Version 2] doi: 10.12688/wellcomeopenres.15405.2 (PMC6790913; doi:10.12688/wellcomeopenres.15405.2)
Supplement: Peer review history

# Peer review: Reviewer response for version 2

**DOI:** 10.21956/wellcomeopenres.17095.r37163

**Published:** 2019-11-26

**Authors:** Sabine Werner, Ulrich auf dem Keller

**Affiliations:** 1 Technical University of Denmark, Kongens Lyngby, Denmark; 2 Institute for Molecular Health Sciences, Department of Biology, ETH Zurich (Swiss Federal Institute of Technology Zürich), Zürich, Switzerland

The authors have appropriately responded to the comments of the reviewers and the manuscript is further improved. The purpose of the publication has been further clarified and I understand that the authors prefer not to include validations into this article. 

 In general, this is a very valuable dataset that will be of interest to researchers in the field of inflammatory skin diseases.

We confirm that we have read this submission and believe that we have an appropriate level of expertise to confirm that it is of an acceptable scientific standard.

# Peer review: Reviewer response for version 1

**DOI:** 10.21956/wellcomeopenres.16840.r36479

**Published:** 2019-10-09

**Authors:** Sanja Kezic

**Affiliations:** 1 Amsterdam UMC, Coronel Institute of Occupational Health, Amsterdam Public Health research institute, University of Amsterdam, Amsterdam, The Netherlands

Elias *et al*. investigated the effect of filaggrin deficiency in an optimized skin organoid model. Next to the inside/out and outside/in barrier function assessed respectively by TEWL and penetration of a hydrophilic dye, a global mass proteomic analysis has been done. As discussed by authors, the used model has important advantage that the changes related to filaggrin deficiency could be studied in specific cells (keratinocytes) and both epidermal and epidermal compartments were present. On the other side, the model misses other relevant cell types. The study is well designed and presented.

 Specific points 

 1. Skin barrier function assessment

 Although Lucifer yellow is a commonly used model penetrant it has to be realized that owing to its hydrophilicity and a large molar mass (444 Da) it has quite low penetration rate and a long lag time. The incubation time of 4 hours before microscopic examination is likely too short to detect penetration across the SC unless the barrier damage is substantial. In general, it would be good to include in functional studies both, hydrophilic and lipophilic penetrants and measure their penetration at different time points. This might provide more detailed insight into alterations in the structure and composition of the SC and relate them to the structural changes. As noted by the authors, the Flg-knock down ‘’reveal various abnormalities: in the lamellar bilayer structure and less prominent corneodesmosomes’’ and furthermore down-regulation of proteins involved in metabolism of lipids’ and ‘phospholipid metabolism’’ and thinner stratum corneum. Though, no changes in either TEWL or dye penetration have been found. This model would be of great value to further study functional consequences of filaggrin deficiency, including previous damage of the skin e.g. by skin irritants. 

 2. Other points

 The authors note that ‘’FLG knockdown in the skin organoids persisted to day 10 after siRNA transfection, as demonstrated by qPCR (Figure 2) and western blotting (Figure 3)’’. In Fig. 2 it is obvious that the knock-down at the RNA level was not complete and lower than achieved in similar studies (van Dongelen *et al*., 2013 ^1^, Wang *et al*., 2017 ^2^). On which day was the mRNA expression measured and what might be reason for quite a low knock-down efficiency?

 Ceramide analysis has been carried out in the model epidermis. Performing ceramide analysis in the isolated stratum corneum might have been more relevant.

I confirm that I have read this submission and believe that I have an appropriate level of expertise to confirm that it is of an acceptable scientific standard.

## References

[1] : Knock-down of filaggrin does not affect lipid organization and composition in stratum corneum of reconstructed human skin equivalents. *Exp Dermatol*.2013;22(12) : 10.1111/exd.12271 807-12 10.1111/exd.12271 24164439

[2] : Deficiency of filaggrin regulates endogenous cysteine protease activity, leading to impaired skin barrier function. *Clin Exp Dermatol*.2017;42(6) : 10.1111/ced.13113 622-631 10.1111/ced.13113 28556377

# Response

**Published:** 2019-11-19

**Authors:** Sara J. Brown

**Affiliations:** University of Dundee, UK

Specific points

*1. Skin barrier function assessment*

**

We agree that Lucifer yellow has a slow penetration rate hence our long incubation (4 hours, compared to some other publications reporting 1 hour or less). We will, in future work, consider use of a hydrophobic dye as an additional, complimentary assessment - thank you for this useful suggestion.

*2. Other points*

The mRNA quantification was carried out 10 days after siRNA transfection and we agree it is interesting to note this decay in knockdown at transcriptome level, whilst the protein knockdown persists to a greater degree. This is likely to reflect the different half-lives of *FLG *mRNA (approximately 2.2 hours ^1^) whereas the half-lives of profilaggrin and filaggrin are approximately 6 and 24 hours respectively. ^2^ We have added this additional explanation to the updated manuscript.

Thank you for the knowledgeable advice about ceramide analysis. We agree that the stratum corneum is the most relevant tissue layer in which to perform lipidomic analysis, but an accurate separation of the whole stratum corneum (without upper epidermal cells) proved to be technically challenging in the organoid model. We therefore chose to analyse full thickness epidermis to ensure consistency between the replicate experiments.

**References**

1.         Nirunsuksiri, W., Zhang, S.H. & Fleckman, P. Reduced stability and bi-allelic, coequal expression of profilaggrin mRNA in keratinocytes cultured from subjects with ichthyosis vulgaris. *J Invest Dermatol* **110**, 854-61 (1998).

2.         Markova, N.G. *et al.* Profilaggrin is a major epidermal calcium-binding protein. *Mol Cell Biol* **13**, 613-25 (1993).

# Peer review: Reviewer response for version 1

**DOI:** 10.21956/wellcomeopenres.16840.r36429

**Published:** 2019-10-01

**Authors:** Neil Rajan

**Affiliations:** 1 Institute of Genetic Medicine, Newcastle University, Newcastle upon Tyne, UK

Elias *et al.* present data on a manuscript titled "Proteomic analysis of a filaggrin-deficient skin organoid model shows evidence of increased transcriptional-translational activity, keratinocyte-immune crosstalk and disordered axon guidance”.

 In this study, primary keratinocytes from 4 female filaggrin wild-type genotyped individuals were subject to siRNA *FLG* kd and a nontargeting siRNA control. These were grown in a skin equivalent model with matched donor fibroblasts, and then keratinocytes alone were harvested after the model had been in at the air liquid interface for 7 days. This was used to carry out proteomic and lipidomic analysis. Additional samples were used for validation work.

 The work has been meticulously described in the methods and is an exemplar for human primary keratinocyte research. Filaggrin knockdown was demonstrated at the transcriptomic and protein level, and is evident in the mass spec data across all samples. A phenotype is seen on TEM and is elegantly presented Fig 5. Barrier function as assessed by TEWL and dye penetration is not impaired following FLG knockdown at Day 7, which is consistent with prior reports using skin equivalent models.

 The key findings are the differentially expressed proteins in this novel model, and unlike prior skin equivalent papers, here dermal fibroblasts are used, allowing for better modelling of human skin where cross talk happens. Notably, lipidomic studies did not show a difference in epidermal ceramides.

 Comments:

1. The authors highlight the proteins that have relevance to the known pathogenetic mechanisms of eczema. It would be helpful to emphasise the novel findings detected in this model compared to the prior proteomic analysis published on filaggrin knockdown keratinocytes (Elias *et al*., 2017 ^1^), as that model did not include matched donor fibroblasts.
2. Table 2 presents Reactome data and highlights Axon guidance, and it would be helpful to see this in the context of the other pathways described. I would favour this being included in the PDF, rather than Fig 9, which may be moved to supplementary data.
3. The volcano plot (Fig 10) is of interest, but more data could be presented here, for example, the 7 points which are log2 fold change >+5 could be identified to make this more informative for the reader. I am uncertain if the colour scheme with p value cut offs is helpful; the authors carefully justify using selected fold change thresholds in the methods section over arbitrary thresholds that are p-value driven and this depiction is at odds with this.
4. Could the title be improved to reflect the more complete nature of this skin equivalent model that incorporates fibroblasts? Also, how were the three elements "increased transcriptional-translational activity, keratinocyte-immune crosstalk and disordered axon guidance” selected or prioritised from the list of Reactome findings?

There is a minor typographical error on page 3. “Medium was refreshed on alternate day sand the cultures were used for analysis up to day 12 (representing three, five and seven days after lifting to the air-liquid interface)".

I confirm that I have read this submission and believe that I have an appropriate level of expertise to confirm that it is of an acceptable scientific standard.

## References

[1] : Proteomic analysis of filaggrin deficiency identifies molecular signatures characteristic of atopic eczema. *J Allergy Clin Immunol*.2017;140(5) : 10.1016/j.jaci.2017.01.039 1299-1309 10.1016/j.jaci.2017.01.039 28479159PMC5667587

# Response

**Published:** 2019-11-19

**Authors:** Sara J. Brown

**Affiliations:** University of Dundee, UK

Comments:

*The authors highlight the proteins that have relevance to the known pathogenetic mechanisms of eczema. It would be helpful to emphasise the novel findings detected in this model compared to the prior proteomic analysis published on filaggrin knockdown keratinocytes (Elias et al., 20171), as that model did not include matched donor fibroblasts.*

Thank you for this insightful comment. The current work differs in several ways from the previous work (performed by the same first author in different research institutions):

(i) The current work used FLG wild-type primary keratinocytes isolated from female breast skin and co-cultured with 3T3 feeder cells in the presence of serum, whereas the previous publication used keratinocytes isolated from foreskin donors and cultured in serum-free conditions.

(ii) The current work used a full-thickness skin organoid model ((Keratinocytes seeded on top of donor matched fibroblasts embedded in a fibrin dermal matrix) whereas the previous work was anepidermal equivalent model (Keratinocyes grown in a transwell) .

(iii) The current work benefited from advancements in proteomic technology and generated a more extensive, detailed proteomic dataset.

The previous work provided, for the first time, evidence that filaggrin deficiency can alter the expression levels of proteins relevant to the pathogenesis of AE, even without an external inflammatory stimulus and the current work supports this.  

Mechanistic themes emerging from this study, including inflammatory, proteolytic and cytoskeletal pathways, broadly overlap with findings from this previous work. However, novel findings detected in the current model compared to the prior proteomic analysis (Elias et al., 2017) may be summarised as follows:

- Evidence that filaggrin-deficient keratinocytes are a source of immune signalling.
- Filaggrin deficiency leads to an increase in transcriptional and translational activity but a reduction in multiple markers of terminal differentiation.
- There is a dysregulation of axon guidance (with elements of this pathway represented in both the up- and down-regulated protein sets).

*Table 2 presents Reactome data and highlights Axon guidance, and it would be helpful to see this in the context of the other pathways described. I would favour this being included in the PDF, rather than Fig 9, which may be moved to supplementary data.*

Thank you for this suggestion. We agree that Table 2 is a valuable summary of the findings from Reactome, but it is too large (including detailed lists of protein IDs, which we are keen to include) to fit into the .pdf. We hope that readers will easily access the full table via the direct link provided to FigShare. We have considered alternative methods to present our Reactome pathway analyses, but the visualisation of pathways within a global over-view does not lend itself to our data where relatively small numbers of pathways (39 and 113) show significant dysregulation.

    

*The volcano plot (Fig 10) is of interest, but more data could be presented here, for example, the 7 points which are log2 fold change >+5 could be identified to make this more informative for the reader. I am uncertain if the colour scheme with p value cut offs is helpful; the authors carefully justify using selected fold change thresholds in the methods section over arbitrary thresholds that are p-value driven and this depiction is at odds with this.*

Thank you for this advice. On further consideration we agree that the colour-coding is not helpful and the addition of labels to the most extreme data points is informative for readers. These changes have been made in a corrected version of Fig 10 in the updated manuscript.

*Could the title be improved to reflect the more complete nature of this skin equivalent model that incorporates fibroblasts? Also, how were the three elements "increased transcriptional-translational activity, keratinocyte-immune crosstalk and disordered axon guidance” selected or prioritised from the list of Reactome findings?*

In response to your helpful suggestion we have added the words ‘full thickness’ to the title to emphasise the nature of our model. Because the title was already very long we have truncated it, since the main findings (‘increased transcriptional-translational activity, keratinocyte-immune crosstalk and disordered axon guidance’) are clearly stated in the abstract.

These three elements were prioritised as noteworthy findings based on a combination of GO and pathway analyses as follows:

(a) Aspects of transcriptional and translational activity are represented in all 10 of the most significant GO terms for Biological process in the consistently down-regulated (Table 1) and up-regulated (Table 3) proteins; ‘Metabolism of RNA’ is the top Reactome term in the down-regulated protein dataset (Table 2) and the Reactome term ‘Translation’ is top in the upregulated proteins (Table 4).  

(b) Keratinocyte-immune crosstalk is highlighted to emphasise that these experiments, in which epidermal keratinocytes are isolated from other cells of the immune system, still generate signals classified within the terms ‘innate’ and ‘acquired immunity’.  In the Reactome analyses of consistently down-regulated proteins 7/39 enriched pathways are related to the immune or antiviral systems and 15/113 enriched pathways in the up-regulated proteins relate to immune systems or antiviral systems.

(c) Disordered axon guidance was represented by only one Reactome term in each of the up- and down-regulated protein groups, but together these account for 74/541 (14%) of the proteins in this pathway. This was prioritised as a novel signal of interest, likely to be relevant to AD pathophysiology but not currently targeted for therapy.

*There is a minor typographical error on page 3. “Medium was refreshed on alternate day sand the cultures were used for analysis up to day 12 (representing three, five and seven days after lifting to the air-liquid interface)".*

Thank you, we have corrected this typo in the updated version of our manuscript.

# Peer review: Reviewer response for version 1

**DOI:** 10.21956/wellcomeopenres.16840.r36427

**Published:** 2019-10-01

**Authors:** Sabine Werner

**Affiliations:** 1 Institute for Molecular Health Sciences, Department of Biology, ETH Zurich (Swiss Federal Institute of Technology Zürich), Zürich, Switzerland

The authors generated and characterized 3D organotypic cultures using keratinocytes with filaggrin knock-down or control keratinocytes. Although such cultures had been generated before, they had not been used for proteomics studies. The proteomics analysis provides some insight into the molecular mechanisms underlying the effect of filaggrin on keratinocyte function. The advantage of this system is the focus on the keratinocyte phenotype. The disadvantage is the lack of the influence of other cell types that are important for Atopic Dermatitis pathogenesis. Overall, this is an interesting study, although there is no functional follow-up of any of the differentially expressed proteins. In addition, the statistical analysis requires improvement.

 Specific comments:

1. The filaggrin knock-down at the RNA level was only approximately 50%, whereas the knock-down at the protein levels seems more robust. How do the authors explain this inconsistency? Why are there no error bars in Fig. 2 NT-siRNA? The data should be confirmed by immunostaining of the cultures for filaggrin. Staining of the cultures for loricrin would be helpful to confirm abnormalities in the granular layer.
2. It is impossible to see obvious differences in Lucifer yellow penetration and there is also no statistically significant difference. This should be expressed more carefully.
3. The proteomics data provide a very important resource. It would have been helpful to show the validation of some of the data using independent samples and another method, e.g. Western blot and/or immunohistochemistry. This would be easy for selected proteins, such as the different keratins.
4. A comparison of the data with available transcriptomics data from AD patients would be interesting.
5. For the proteomics analyses, it is not clear how the false discovery rate was exactly determined (scrambled or reversed database?) and how false positives were removed. This requires clarification. 
6. Statistical analysis to identify proteins with differential abundance between conditions lacks stringency and appears asymmetric with regard to positive and negative effect sizes. Lack of statistically significant hits upon multiple testing correction is a common problem with proteomics datasets. Which multiple testing correction algorithm was applied? Bonferroni or Benjamini-Hochberg? Did the authors try e.g. a limma moderated t-test that has proven useful for proteomics data? Even without multiple testing correction, a statistical test appears more suitable than selecting differentially abundant proteins only based on consistency between comparisons/experiments. Moreover, the authors apply a cut-off of 2-fold (0.5) to identify proteins with lower abundance upon filaggrin knockdown and of 1.2-fold (1.2) for those with higher abundance in this condition. It appears this skewed effect size has been selected aiming at equalizing hit numbers for pathway enrichment. This should be reconsidered. The terms ‘ratio’ and ‘fold-change’ are not used properly, since the authors report actual ratios as fold-changes, e.g. a ratio of 0.5 would correspond to a fold-change of -2 and of 2 to a fold change of +2. Since identifying proteins with statistically significantly differential abundance between conditions is crucial, also for follow-up data analysis, the authors should re-evaluate this part of the study with help of an expert in proteomics statistics.

I confirm that I have read this submission and believe that I have an appropriate level of expertise to confirm that it is of an acceptable scientific standard, however I have significant reservations, as outlined above.

# Response

**Published:** 2019-11-19

**Authors:** Sara J. Brown

**Affiliations:** University of Dundee, UK

Thank you for your interest in the skin organoid model and our analyses. We believe that a focus on the keratinocyte phenotype is valuable, particularly in AD where the keratinocyte’s contribution to barrier function is of particular interest. Whilst we agree that there are many other cell types contributing to AD pathogenesis, we consider that the isolation of keratinocytes may be viewed as an advantage in this experimental system because it provides an opportunity to investigate the specific contribution of keratinocyte biology to features of atopic inflammation and other pathomechanisms in this highly complex trait.

We agree that functional follow-up may be an important step and we have conducted extensive validation in other applications of the skin organoid and a simpler epidermal model. ^1,2^ The detailed dataset and our analyses reported here in *Wellcome Open Research* are shared as a resource for the community, to allow others to review the differentially expressed proteins and follow up selected candidates as indicated. We have further clarified this as the principle aim of our publication in Version 2.

Specific comments:

*The filaggrin knock-down at the RNA level was only approximately 50%, whereas the knock-down at the protein levels seems more robust. How do the authors explain this inconsistency?*

We agree, there was a 0.5-fold reduction in *FLG* mRNA compared to 0.36- and 0.25-fold reduction in pro-filaggrin and filaggrin proteins respectively. These differing levels of reduction in expression reflect the use of siRNA which creates a transient knockdown. siRNA is our preferred methodology because we aim to maintain the primary cells at low passage and to minimise non-physiological experimental influences. The different degrees of knockdown reflect the long experimental time points (10 days post transfection for a mature organoid) and the inherent differences in mRNA and protein stabilities. *FLG *mRNA has a reported half-life of ~2.2 hours ^3^ whereas the half-lives of profilaggrin and filaggrin are approximately 6 and 24 hours respectively. ^4^ We have added this additional explanation to the updated manuscript. Since our main experimental aim was to reduce filaggrin at the protein level, we are satisfied that knockdown of the functional monomer is maintained for the duration of our experiment and this degree of reduction is in keeping with the reduction in expression observed in *FLG*-null heterozygotes or atopic skin.

*Why are there no error bars in Fig. 2 NT-siRNA?*

The filaggrin expression presented in Figure 2 has been calculated in a pair-wise manner ( *FLG*-siRNA versus *NT*-siRNA control) for each independent biological donor (N=8) and the replicate data were then combined for statistical analysis. Since only one technical replicate was performed per donor, the *NT*-siRNA condition is calculated as 1 in each biological replicate and hence displays no error in the combined dataset. This internally controlled method of analysis accounts for differences in baseline filaggrin expression and technical variations between replicate experiments.

*The data should be confirmed by immunostaining of the cultures for filaggrin.*

Thank you for this suggestion. We agree that immunostaining can represent a valuable confirmatory approach, however the H&E stained section show a clear reduction in the granular layer and the filaggrin knockdown at protein level was confirmed by mass spec proteomics (mean 0.27-fold, N=4) which is likely to be more sensitive for the detection of quantitative differences than immunostaining.

*Staining of the cultures for loricrin would be helpful to confirm abnormalities in the granular layer.*

The electron microscopy (Figure 5) shows the granular layer in some detail, with a clear reduction in the number and size of granules. We therefore did not elect to stain the organoid cultures for loricrin specifically.

    

*It is impossible to see obvious differences in Lucifer yellow penetration and there is also no statistically significant difference. This should be expressed more carefully.*

Thank you for bringing this to our attention. We did observe a difference in Lucifer yellow dye penetration in 3/5 of the biological replicates at day 3 but we agree this is not apparent in the mature model at day 7 (example images shown in Figure 8A). We aimed to explain that our quantification (in Figure 8B) shows all of the individual data points and, whilst some experiments showed an increase in dye penetration in the immature organoid, we state “However, this delay in maturation of the ‘outside-to-inside’ barrier function was not consistently demonstrated in all biological replicates.” In light of your comment we have added further clarification to the text.

    

*The proteomics data provide a very important resource. It would have been helpful to show the validation of some of the data using independent samples and another method, e.g. Western blot and/or immunohistochemistry. This would be easy for selected proteins, such as the different keratins.*

Thank you, we agree the proteomics data are a valuable resource and we are delighted to share this resource. We also agree it will be relatively easy for other groups to validate proteins of interest in their work; therefore, rather than carrying out validation ourselves for selected keratins (or multiple different proteins as we have for our parallel proteomics analyses, recently published ^1^) we prefer to share the data for further independent validation.

*A comparison of the data with available transcriptomics data from AD patients would be interesting.*

We agree that this would be a very interesting analysis; it is not within the remit of this study but we or others may pursue this in the future.

*For the proteomics analyses, it is not clear how the false discovery rate was exactly determined (scrambled or reversed database?) and how false positives were removed. This requires clarification.*

We apologise for the lack of clarity in this description. The false discovery rate was determined using a reversed database. The peptide and protein false discovery rate was set at 1%, and these identified peptides were denoted with REV_ and removed from further analysis. These details have been added to the revised manuscript.

    

*Statistical analysis to identify proteins with differential abundance between conditions lacks stringency and appears asymmetric with regard to positive and negative effect sizes. Lack of statistically significant hits upon multiple testing correction is a common problem with proteomics datasets. Which multiple testing correction algorithm was applied? Bonferroni or Benjamini-Hochberg? Did the authors try e.g. a limma moderated t-test that has proven useful for proteomics data? Even without multiple testing correction, a statistical test appears more suitable than selecting differentially abundant proteins only based on consistency between comparisons/experiments.*

We appreciate that there are no clear guidelines on methodology for the analysis of large proteomics datasets (or indeed many other large datasets) and different expert opinions exist regarding the most appropriate tools and thresholds to define ‘significance’. We have benefited from the considerable expertise within our multidisciplinary collaboration, leading to the rather simple data analysis that we have performed, whilst providing the full dataset for others to analyse as they may wish.

A full Bonferroni correction does not identify any differentially expressed proteins because of the large number detected in this global analysis. Because of the nature of our data, generated using biological replicate samples (primary cells from 4 separate human donors), and the focus on a common complex trait, we prefer to use the criterion of *consistency* across the biological replicates, with filtering for fold change to provide similar numbers of up- and down- regulated proteins, followed by network and pathway analyses.

**

*Moreover, the authors apply a cut-off of 2-fold (0.5) to identify proteins with lower abundance upon filaggrin knockdown and of 1.2-fold (1.2) for those with higher abundance in this condition. It appears this skewed effect size has been selected aiming at equalizing hit numbers for pathway enrichment. This should be reconsidered.*

Thank you for highlighting this question. We have stated clearly and openly that we *did* select these cut-offs aiming to equalize protein numbers in this right-skewed dataset. Using the thresholds of £0.5 and ³1.2 defines 1422 (~25%) and 1871 (~34%) of the total number of proteins. This is an established approach in proteomic analysis; we appreciate other valid approaches can be applied and we welcome further interrogation of our experimental data. We have further clarified this in the discussion.

*The terms ‘ratio’ and ‘fold-change’ are not used properly, since the authors report actual ratios as fold-changes, e.g. a ratio of 0.5 would correspond to a fold-change of -2 and of 2 to a fold change of +2.*

We apologise for the inconsistency in this terminology and we have corrected this detail throughout the manuscript.

**References**

1.         Elias, M.S. *et al.* EMSY expression affects multiple components of the skin barrier with relevance to atopic dermatitis. *J Allergy Clin Immunol* **144**, 470-481 (2019).

2.         Elias, M.S. *et al.* Proteomic analysis of filaggrin deficiency identifies molecular signatures characteristic of atopic eczema. *J Allergy Clin Immunol* **140**, 1299-1309 (2017).

3.         Nirunsuksiri, W., Zhang, S.H. & Fleckman, P. Reduced stability and bi-allelic, coequal expression of profilaggrin mRNA in keratinocytes cultured from subjects with ichthyosis vulgaris. *J Invest Dermatol* **110**, 854-61 (1998).

4.         Markova, N.G. *et al.* Profilaggrin is a major epidermal calcium-binding protein. *Mol Cell Biol* **13**, 613-25 (1993).
